# Microstructure and Texture Evolution of Cu-Ni-P Alloy after Cold Rolling and Annealing

**DOI:** 10.3390/ma17112696

**Published:** 2024-06-03

**Authors:** Wendi Yang, Chengzhi Zhang, Nan Zhang, Chucan Zhang, Weilin Gao, Jilin He

**Affiliations:** 1 School of Material Science and Engineering, Zhengzhou University, Zhengzhou 450001, China; wdyang@gs.zzu.edu.cn (W.Y.); zhangnanzzu2021@163.com (N.Z.); zhangchucan@gs.zzu.edu.cn (C.Z.); hejilin@zzu.edu.cn (J.H.); 2 Zhongyuan Critical Metals Laboratory, Zhengzhou 450001, China

**Keywords:** Cu-Ni-P alloy, cold rolling, recrystallization, texture

## Abstract

The microstructure and texture evolution of Cu-Ni-P alloy after cold rolling and annealing at 500 °C was studied by electron backscattering diffraction (EBSD). The equiaxed grain is elongated and the dislocation density increases gradually after cold rolling. The grain boundaries become blurred and the structure becomes banded when the reduction in cold rolling reaches 95%. A typical rolling texture is formed with the increase in deformation amount in cold rolling. The deformation structure gradually disappeared and recrystallized new grains were formed after annealing at 500 °C. The recrystallization nucleation mechanism of Cu-Ni-P alloy at 60% reduction is mainly a bow nucleation mechanism. A shear band begins to form after annealing at 80% reduction. The shear band becomes the preferred nucleation location with the increase in reduction. Most adjacent recrystallized grains growing in the shear band have a twin relationship.

## 1. Introduction

Copper alloys are favored due to their excellent thermal and electrical conductivity, good plasticity and outstanding corrosion resistance among other characteristics. They are widely used in electric power, electronic information, machinery manufacturing, transportation, defense and aerospace [1,2,3]. Copper alloys with an excellent balance of strength and conductivity are needed to meet the needs of electric vehicles, fast charging, high-speed transmission, etc. [4,5,6].

The conductivity of Cu-Cr-Zr alloy can reach 80% IACS, but the strength is slightly insufficient. The strength of Cu-Ni-Si alloys is up to 950 MPa, but the conductivity is lower than 60% IACS [1,5]. Precipitation-strengthened Cu-Ni-P alloys have received much attention and are expected to meet the performance requirements of high strength and high conductivity in recent years. Cu-Ni-P alloy can form a Ni-P precipitated phase, so the residual solid solution of the Ni and P in the Cu matrix is reduced to a very low degree, in addition to the excellent stress relaxation properties. Therefore, Cu-Ni-P alloys have the potential for mechanical properties of high strength and high electrical conductivity. Ni_5_P_2_ was discovered by Crampton [7], Ni_12_P_5_ was discovered by Aruga [8], Ni_5_P_4_ was discovered by Murayama [9] and Ni_2_P was discovered by Hu [7] and have been confirmed as typical precipitates of Cu-Ni-P alloys among Cu-Ni-P alloys after aging. Massive literature have also reported the method of adding Fe [10], Zr [11], Sn [12] and other trace elements in Cu-Ni-P alloy to improve the comprehensive properties of Cu-Ni-P alloy. However, it often leads to poor bending processability properties of the alloy while increasing its strength. It is reported that there is a close relationship between bending processability and texture [13,14,15]. The microstructure and texture evolution of copper alloys after cold rolling deformation and annealing have been reported in recent years. Belyakov [16] has studied the deformation microstructure of pure copper and Cu-1.5%Ni-0.3%P alloys during large-strain plastic processing. The results show that the texture involves most of the “brass” {011}<211> texture components in the studied strain range. The samples aged at 600 °C showed continuous strain hardening during cold working. Twinning and micro-shearing are significantly delayed, and the final texture has no unique preferred orientation. Zhu et al. [17] prepared a Cu-0.96Ni-0.22P alloy with a good balance of strength and conductivity, and studied its microstructure evolution during processing in detail. The research results showed that the fibrous grain is formed along the rolling direction after 60% reduction, and the grain structure is further refined at 80% reduction. The increase in the deformation storage energy accelerates softening during aging. Lin et al. [18] studied the microstructure and texture evolution of Cu-Mn alloy during static recrystallization under different reduction amounts. The results showed that the maximum value of the texture shifted from the S orientation and the Goss orientation to the Cube orientation, and from the brass orientation to the BR orientation under the same annealing conditions, with the increase in reduction. Hu et al. [19] show that high rolling temperatures favor the copper texture and low rolling temperatures favor the brass texture. Leffers [20] shows that a low strain rate favors a copper texture, while a high strain rate favors a brass texture. However, the microstructure and texture evolution of Cu-Ni-P alloy during cold rolling and annealing are still few and need to be studied more systematically.

Therefore, the precipitation strengthened Cu-0.6Ni-0.158P (CNP) alloy was prepared by vacuum melting. Four rolling reductions of 60%, 80%, 90% and 95% were selected to systematically investigate the microstructure and texture evolution of Cu-Ni-P alloy during cold rolling and the texture evolution of Cu-Ni-P alloy after annealing at different rolling reductions. It is hoped that it will provide the basis for texture control of Cu-Ni-P alloy.

## 2. Experimental

### 2.1. Material and Preparation

**Figure 1:**
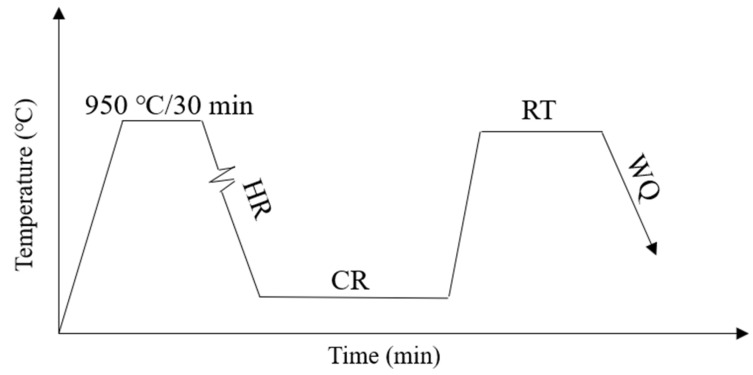
Detailed processing flow diagram of the studied Cu-0.6Ni-0.158P (CNP) alloy.

The raw material (99.9% pure copper sheet (Tongling Nonferrous Metals, Tongling, China), Cu-14P master alloy (Tongling Nonferrous Metals, China), 99.99% Ni sheet (Zhongyan metal materials Ltd., Tianjin, China)) was used as a master batch that melted in the vacuum induction furnace under an Ar atmosphere. The specific composition of the alloy was detected four times by an inductively coupled plasma optical emission spectrometer (ICP-OES) (shimao2u, Kyoto City, Japan). The composition of ingots is listed in Table 1. The content of Ni and P elements has a great influence on the Electrical conductivity. So the conductivity can reach more than 60% IACS while obtaining high strength when the content of Ni and P is designed. The added element content can save costs in actual production at the same time. Figure 1 shows the subsequent typical process flow of the ingot obtained by smelting and casting.

Homogenization (950 °C, 30 min) → Hot rolling (HR) → Cold rolling (60%, 80%, 90%, 95%) → Recrystallization treatment (RT, 500 °C/1 min, 5 min, 30 min, 60 min, 240 min, 480 min, 600 min) → Water quenching (WQ).

### 2.2. Methods

The microhardness was tested five times on an HV-0.3 micro-Vickers loading of 300 g with a dwell time of 10 s. The standard deviation of the error bar is 0.1. The crystallographic features of local orientations were characterized by means of electron back scattering diffraction (EBSD) using an Oxford EBSD camera and the AZtec online acquisition software package (AZtecCrystal 2.1, Oxford Instruments Nanotechnology Tools Ltd., Oxford, UK). The EBSD maps presented in this paper are depicted by inverse pole figure (IPF) maps indicating the ND (Normal Direction, ND), RD (Rolling Direction, RD), and TD (Transverse Direction, TD) in the crystal reference frame. The specimen was first mechanically polished and followed by electrolytically polished with a solution of H_3_PO_4_ + CH_3_OH + H_2_O with a volume ratio of 1:1:2. The dislocation density was detected by Empyrean X-ray diffractometer (PANalytical, Almelo, Netherlands), with the scanning range varying from 10 to 90 deg at a step size of ~0.02 deg and a scanning velocity of ~4 deg/min. The ray source is Cu-Kα radiation. The specimen was mechanically polished to a clean surface with no obvious scratches. TEM analysis was performed on a FEI/Talos F200S (FEI Czech Ltd., Černovice, Czech Republic) transmission electron microscope with an accelerated voltage of 200 kV. The TEM specimen was first mechanically polished to a thickness of about 50 μm. A Leica EM Res102 multi-function ion thinning instrument (Leica, Wetzlar, Germany) was used for thinning treatment.

## 3. Result

### 3.1. The Evolution of Deformed Structure

**Figure 2:**
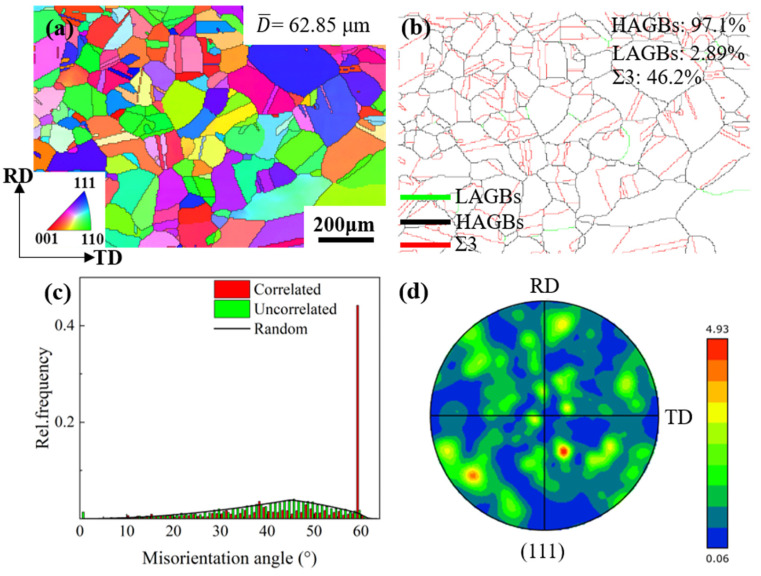
EBSD map of Cu-Ni-P alloy in hot rolled state: (**a**) IPF/ND map; (**b**) grain boundary distribution map; (**c**) misorientation angle map; (**d**) (111) pole figure.

Figure 2 shows the orientation map of the initial microstructure of Cu-Ni-P alloy after hot rolling (before cold rolling). It can be observed from Figure 2a that the microstructure of Cu-Ni-P alloy is equiaxed grain with a grain size of about 62.85 μm. It can be seen from Figure 2b that HAGBs (High Angle Grain Boundaries, HAGBs) are dominant, accounting for 97.10%. LAGBs (Low Angle Grain Boundaries, LAGBs) accounted for only 2.89% and Σ3 grain boundary accounted for 46.20%. It can be seen from the misorientation angle distribution of initial random orientations in Figure 2c that the trends of "uncorrelated" and "random" curves were similar, indicating that the overall texture strength of hot-rolled tissues was weak. Hence, it could be approximated as a random texture distribution. The (111) pole figure strength in Figure 2d is 4.93, which further indicates that there is no obvious preferred orientation in the hot-rolled structure. The twins in the hot rolling process are mainly annealing twins. Annealing twins are mainly formed in the recrystallization annealing process of deformed metals and are usually bounded by parallel twin planes across the entire grain. The annealing twin is basically inside the grain, through the whole grain, and the twin layer is clearly visible [21].

**Figure 3:**
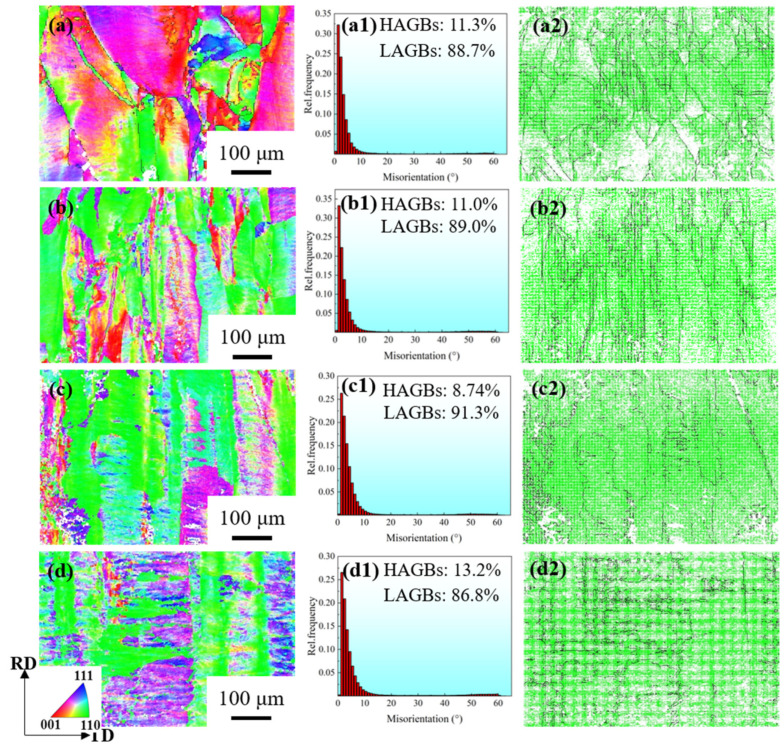
Orientation distribution map of Cu-Ni-P alloy: (**a**–**a2**) 60%; (**b**–**b2**) 80%; (**c**–**c2**) 90%; (**d**–**d2**) 95%; (**a**–**d**) IPF//ND; (**a1**–**d1**) Misorientation distribution map; (**a2–d2**) Grain boundary distribution map.

Figure 3 shows the distribution of the grain boundary and the grain boundary misorientation of Cu-Ni-P alloy with different cold rolling reductions. Massive unevenly distributed LAGBs appear in the alloy microstructure, and the proportion of HAGBs is only 11.3% at 60% reduction. The large grains began to break into small grains, and the grains were gradually elongated, and the proportion of LAGBs with uneven distribution in the alloy structure further increased to 89.0% at an 80% reduction. The grain is further elongated along the rolling direction and gradually develops into a fibrous structure at 90% reduction. The distribution of LAGBs tends to be homogenized, and the area with severe deformation presents a disorderly flocculent distribution, indicating a high defect density, as shown in Figure 5c. All the large grains are broken into small grains. The LAGBs are evenly distributed in the deformed structure at a 95% reduction. The proportion of LAGBs decreased when the reduction was 95% compared with that of 90%. The reason is that the large grains split during the cold rolling process, resulting in a new geometric necessity dislocation boundary (GNB) and an epigenetic dislocation boundary, which are LAGBs when the deformation is small. However, GNB will gradually turn to a rolling direction with the rotation of grains, and the misorientation will further increase, forming HAGBs, which leads to an increase in the proportion of HAGBs under large deformation [22].

**Figure 4:**
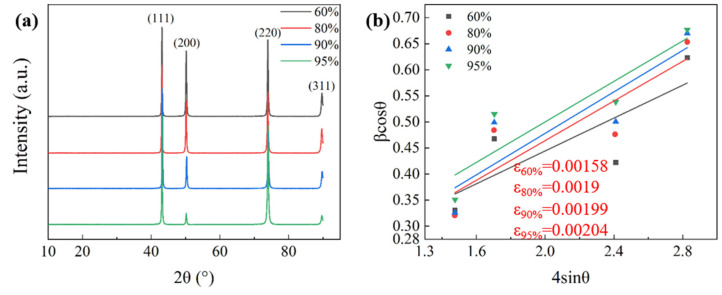
XRD patterns of Cu-Ni-P alloys at different rolling reductions: (**a**) XRD patterns; (**b**) Linear fitting plot of the alloy.

The XRD images of the alloys at different rolling reductions are shown in Figure 4a. The strength of the <110> peak increases with an increase in the rolling reduction. According to the Williamson-Hall equation [23], the micro-strain in the alloy can be reflected by X-ray diffraction data.

(1) $$\beta \cos(\theta )=\frac{k\lambda }{d}+4\varepsilon \sin(\theta )$$

where *β* is the half-peak width of the maximum diffraction peak, *λ* is the radiation wave-length (*λ* = wavelength of Cukα radiation = 1.5406 Å), *k* is a constant of 0.9 (shape factor), *θ* is the Bragg Angle, and *d* is the grain size. Figure 4b shows the 4sin(θ)-βcos(θ) images of the alloy at different rolling reductions. According to Equation (1), the linear fitting of the experimental data shows that the slopes are 0.0882, 0.1159, 0.1144, and 0.0704, respectively.

Therefore, the dislocation density of the alloy can be calculated from the total micro-strain *ε* [24].

(2) $$\rho =\frac{16.1\times \varepsilon^{2}}{b^{2}}$$

where *b* is the Burgers vector equal to √2a/2, takes 0.255 nm, and *a* is the lattice constant of the copper alloy [25]. Therefore, the dislocation densities under different alloy states are calculated as 6.18 × 10^14^/m^2^, 8.94 × 10^14^/m^2^, 9.81 × 10^14^/m^2^, and 10.30 × 10^14^/m^2^, respectively. The results show that the internal dislocation of the alloy increases rapidly with an increase in the rolling reduction.

**Figure 5:**
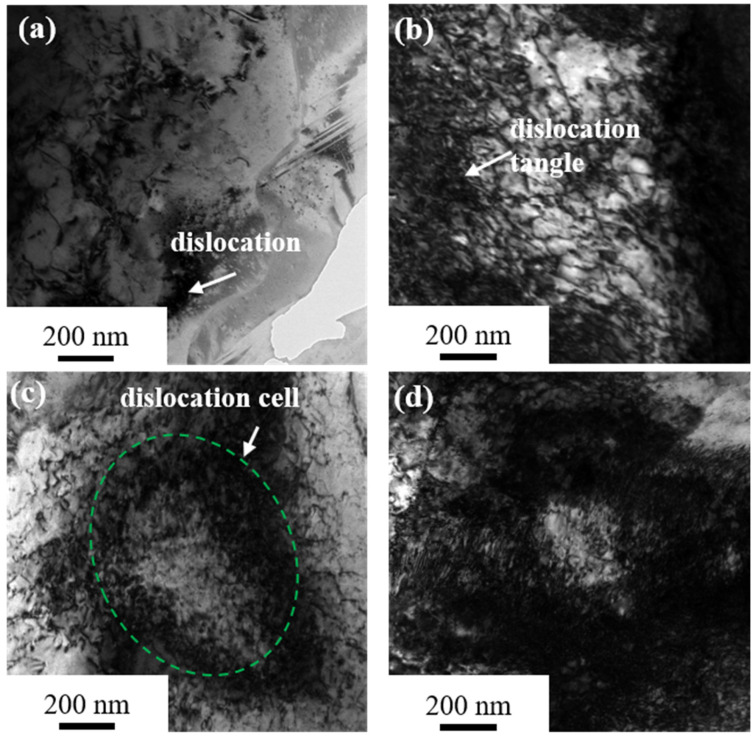
TEM image of Cu-Ni-P alloy under different reductions: (**a**) 60%; (**b**) 80%; (**c**) 90%; (**d**) 95%.

Figure 5 shows typical TEM images of Cu-Ni-P alloy samples deformed by a 60%, 80%, 90% and 95% reduction, respectively. A small amount of dislocation entanglement appears in the microstructure at a 60% reduction. The internal deformation of the material is not uniform, and the dislocation distribution is not uniform due to the low reduction (Figure 5a). The dislocation density increases significantly (Figure 5b), which is the result of the proliferation of movable dislocation with the increase in deformation at an 80% reduction. Figure 5c shows the typical microstructure of the alloy at a 90% reduction, and cellular structure has been formed in some regions. Figure 5d shows the microstructure of the alloy at a 95% reduction. It can be seen that the dislocation density further increases and massive dislocation cells are formed.

**Figure 6:**
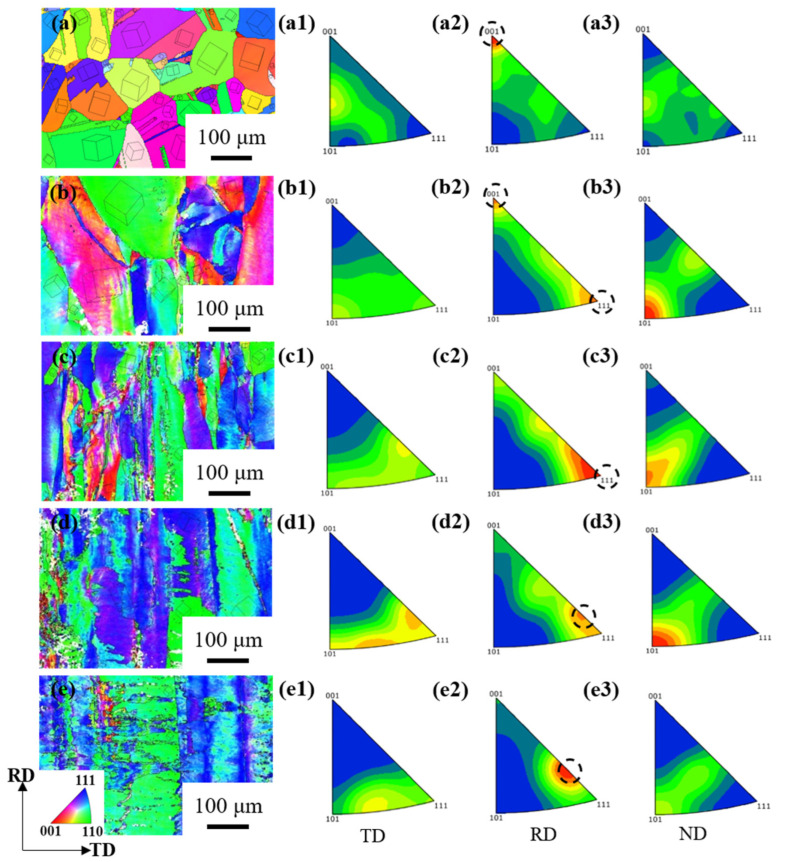
Orientation map of Cu-Ni-P alloy at different rolling reductions: (**a**,**a1**–**a3**) hot rolling; (**b**,**b1**–**b3**) 60%; (**c**,**c1**–**c3**) 80%; (**d**,**d1**–**d3**) 90%; (**e**,**e1**–**e3**) 95%; (**a**–**e**) IPF//RD map; (**a1**–**e1**) IPF//TD; (**a2**–**e2**) IPF//RD; (**a3**–**e3**) IPF//ND.

It can be seen that the texture type is mainly RD//<001> from the IPF//RD of hot-rolled Cu-Ni-P alloy (Figure 6(a2)). The grain begins to slide along {111}<110>, and gradually transitions from RD//<001> to RD//<111> at a 60% reduction. The texture type is RD//<111>, and λ is 90° at an 80% reduction. The Schmidt factor is almost zero, at which point it is relatively difficult to continue sliding. Further analysis shows that the texture {112}<111> is basically formed when RD//<111>. The texture type was mainly RD//<111> but began to transition to RD//<112> when the reduction increased from 80% to 90%. Massive grains cannot maintain the original crystal orientation and continue to deform along the direction of cold rolling, and most of the grains have severe deformation when the reduction reaches 95%. An approximate RD//<112> orientation is eventually formed, that is, a brass texture {110}<112> is formed (Figure 6(e2)).

**Figure 7:**
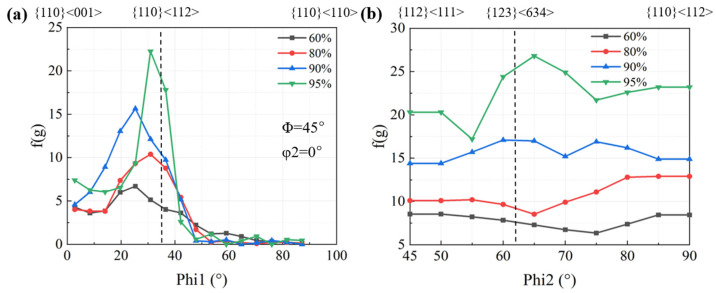
Orientation density f(g) of cold rolled Cu-Ni-P alloy: (**a**) α-fiber; (**b**) β-fiber.

Figure 7 shows the evolution of α-fiber and β-fiber texture of Cu-Ni-P alloy with the increase in rolling reduction. It can be seen from Figure 7b that the texture strength increases uniformly throughout the β-fiber with the increase in rolling reduction. The maximum orientation strength is located at φ2 = 65°, reaching 26.8 at a 95% reduction. The peak value of texture strength increases on α-fiber texture with the increase in rolling reduction. The strength of Goss and Brass textures increases gradually with the increase in rolling reduction, while the development of other textures is obviously asymmetrical. The texture strength decreases sharply when φ1 exceeds 45°.

**Figure 8:**
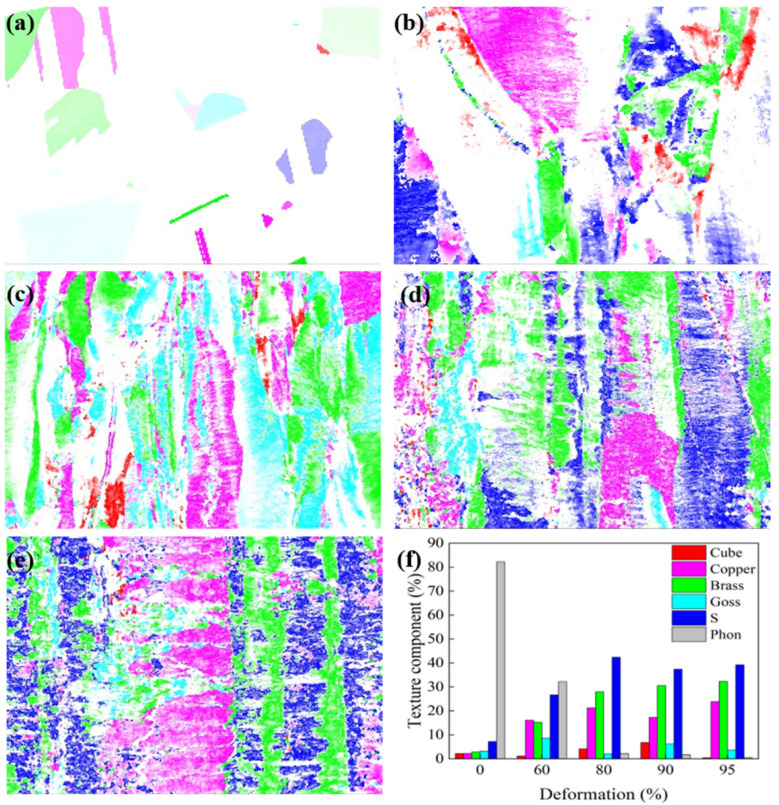
Orientation distribution of Cu-Ni-P alloy under different reductions and the change in the orientation component volume fraction with deformation: (**a**) hot rolling; (**b**) 60%; (**c**) 80%; (**d**) 90%; (**e**) 95%; (**f**) texture component statistical diagram (red: Cube texture; pink: Copper texture; green: Brass texture; light blue: Goss texture; dark blue: S texture; white: phon texture).

The orientation distribution map of Cu-Ni-P alloy under different rolling reductions and the change in the orientation component volume fraction with reduction are shown in Figure 8. The alloy is mainly affected by the normal pressure of the rolling surface and the shear force of the rolling direction in the process of cold rolling. It can be seen from the orientation distribution map under each rolling reduction that the random orientation gradually decreases from the initial 82.2%, forming a typical rolling texture, and massive S-shear texture orientations appear on the rolled surface with the increase in rolling reduction. The dislocation slips and the grain orientation rotates accordingly, concentrating towards {112}<111> or {110}<112>, finally, forming a stable Copper and Brass texture during the deformation process. Because the grain rotation in the direction of {110}<112> requires a large resolved shear stresses, which is difficult to achieve in ordinary cold rolling reduction. Therefore, the grains tend to rotate to the {110}<112> direction, forming a Brass texture only under large reduction. {110}<001> orientation also appears in the alloy, that is, the Goss texture is formed under the condition that the rolling deformation geometry is less limited to the rolling plastic flow [26].

### 3.2. Microstructure Evolution after Annealing at Different Rolling Reduction

**Figure 9:**
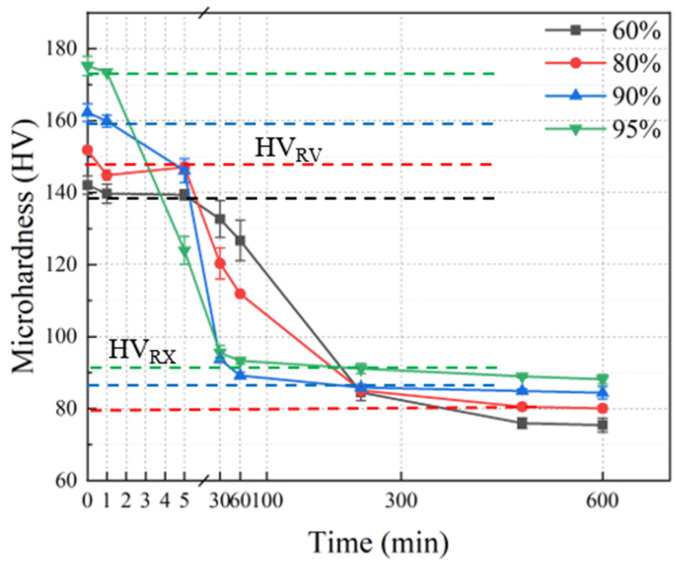
Recrystallization process microhardness curve (black dashed lines: 60% reduction; red dashed lines: 80% reduction; blue dashed lines: 90% reduction; green dashed lines: 95% reduction).

**Figure 10:**
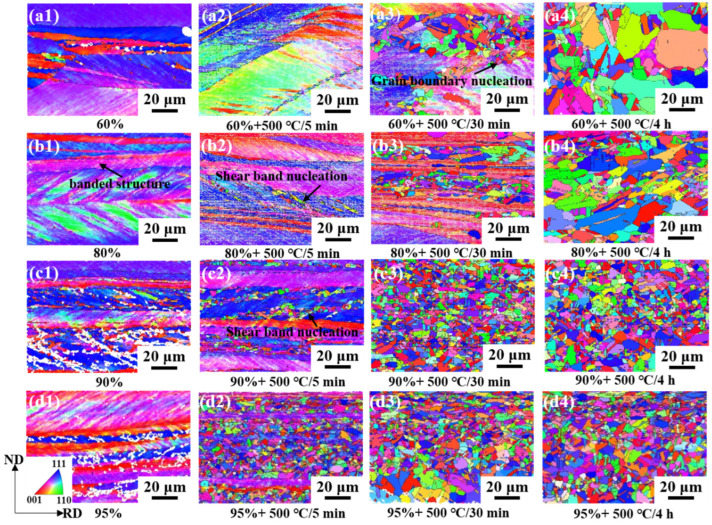
Orientation map of samples with different rolling reduction and annealing time at 500 °C (IPF//RD): (**a1**–**a4**) 60% CR and annealing for 5 min, 30 min, 4 h; (**b1**–**b4**) 80% CR and annealing for 5 min, 30 min, 4 h; (**c1**–**c4**) 90% CR and annealing for 5 min, 30 min, 4 h; (**d1**–**d4**) 95% CR and annealing for 5 min, 30 min, 4 h.

Figure 9 shows the relationship between microhardness and time of Cu-Ni-P alloy with different rolling reductions after annealing at 500 °C for different times. The microhardness value before annealing increases with the increase in the cold rolling reduction due to work hardening. Then, the sample softens when the rolled sample is annealed. The microhardness of the sample decreases rapidly at the beginning; the decreasing rate of microhardness slows down with the increase in annealing time. The average microhardness decreases from 142.1 HV (cold rolled state) to 75.4 HV (500 °C/10 h) with the increase in annealing time when 60% cold rolled samples are annealed at 500 °C. Similarly, the average microhardness of 80% cold rolled samples decreased from 151.8 HV to 80.1 HV with the increase in annealing time. The average microhardness of 90% cold rolled samples decreased from 162.3 HV to 84.4 HV. The 95% cold rolled samples were completely recrystallized (Figure 10d3), and the average microhardness value decreased from 175.2 HV to 88.1 HV after annealing at 500 °C for 30 min. The dashed lines in different colors in Figure 9 represent the positions at the end of recovery (HV_RV_) and recrystallization (HV_RX_) of Cu-Ni-P alloy with different rolling reductions. It can be clearly seen from Figure 9 that the recrystallization rate is faster with the increase in the rolling reduction.

**Figure 11:**
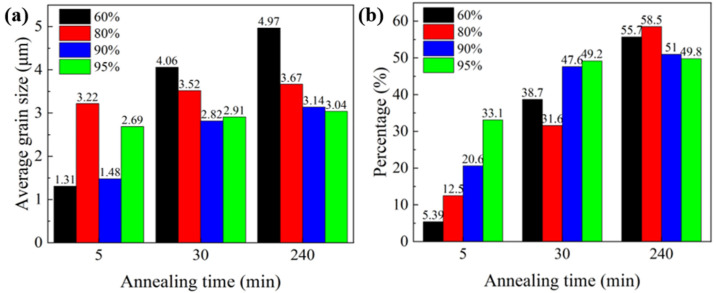
Quantitative statistics of grain information under different annealing conditions: (**a**) grain size of annealed specimen; (**b**) percentage of annealing twins.

Figure 10 shows an orientation map of the samples with different rolling reductions and subsequent annealing. Figure 11 shows the corresponding average grain size and proportion of the annealing twin boundary for the annealed sample. The rolling deformation results in a straight strip microstructure consisting of original grains that are highly elongated along the RD. The sample with a 60% reduction has uneven deformation, with bands along RD. The local deformation is serious and some cracked grains are formed. No obvious recrystallization occurred in the samples at a 60% reduction after annealing for 5 min. Massive recrystallized grains were formed mainly at the grain boundaries after annealing for 30 min. According to the deformation degree and nucleation position, the recrystallization nucleation mechanism of Cu-Ni-P alloy at this time is arch nucleation. The average grain size increases to about 4.97 μm (Figure 11a), and the grain size is not uniform after annealing for 4 h. Shear bands were formed at an 80% reduction, and recrystallization occurred preferentially after annealing for 5 min. The deformation structure gradually disappeared with the increase in the annealing time. The average grain size of the equiaxed recrystallized grains reaches 3.67 μm (Figure 11a), and massive twins continuously appear inside the grains by increasing the annealing time to 4 h. The grain develops into a thin ribbon structure, forming massive shear bands at a 95% reduction. The recrystallization rate is further increased due to large deformation stores with high-strain energy. Massive recrystallized grains were formed compared with the 60%, 80% and 90% rolled samples after annealing for 5 min. The deformation structure almost completely disappeared and equiaxed recrystallized grains are formed when the annealing time is extended to 30 min. Some of the recrystallized small grains engulf other grains, forming larger equiaxed grains in which twins are clearly visible, with an average grain size of 3.52 μm (Figure 11a). The average grain size changed little, about 3.67 μm when the annealing time increased to 4 h.

**Figure 12:**
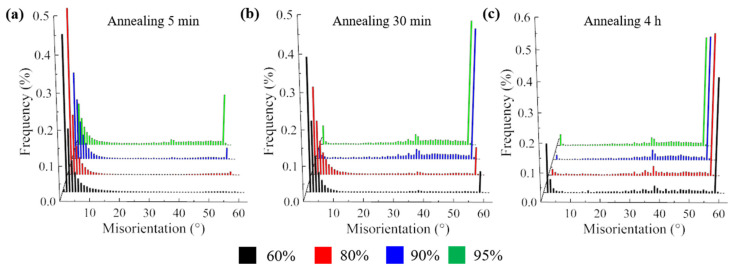
Grain boundary misorientation distribution of samples annealed at 500 °C for different time: (**a**) annealing for 5 min; (**b**) annealing for 30 min; (**c**) annealing for 4 h.

Figure 12 shows the grain boundary misorientation distribution of Cu-Ni-P alloys with different rolling reductions annealed at 500 °C at different times. The misorientation of the annealed sample with a 60% rolling reduction is still dominated by the LAGBs, accounting for 86.2% after annealing for 5 min. The annealing samples with 80% and 90% rolling reduction have a 60° twin boundary. The annealing samples with a 95% rolling reduction have a massive twin boundary with a misorientation at 60°, indicating that the twin has begun to form. The proportion of LAGBs decreased while the proportion of HAGBs increased after annealing for 30 min. Small peaks of 60° were observed in annealed samples with a 60% and 80% rolling reduction and distinct peaks of 60° were observed in annealed samples with a 90% and 95% rolling reduction. In addition to the peak at 60°, a second peak occurs at about 40°. LAGBs at a 95% rolling reduction have basically disappeared, indicating that the recrystallization has been basically complete when the annealing time is increased to 4 h. The distribution of HAGBs is random, but it still occupies the highest proportion at the 60° annealing boundary. Most HAGBs are between 30° and 55°, with secondary peaks still distributed at 40°. The 60° misorientation is mainly caused by the fact that most of the newly generated recrystallized grains maintain a common twin orientation relationship with the matrix (Σ3 grain boundary), and massive twins exist in the annealed structure. Duggan [27] shows that grain boundaries of 40°<111> have the greatest mobility, which leads to “micro-growth selection”.

## 4. Nucleation Site Analysis

**Figure 13:**
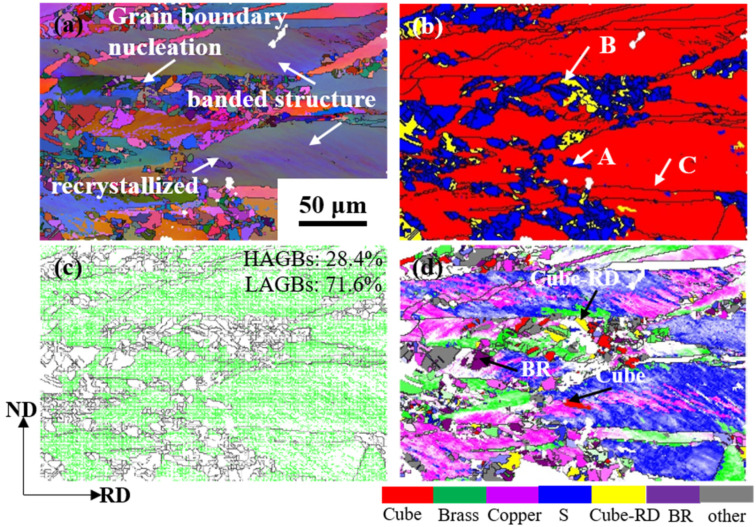
Orientation map of Cu-Ni-P alloy with 60% rolling reduction after annealing at 500 °C for 30 min: (**a**) Full Euler angle map; (**b**) Recrystallization orientation map; (**c**) Grain boundary map; (**d**) Nucleation location map.

To further elucidate the mechanisms of recrystallization nucleation, the nucleation of recrystallization grain orientation at grain boundaries and shear bands is analyzed. Figure 13 shows the orientation map of the Cu-Ni-P alloy sample with a 60% reduction at 500 °C/30 min. It can be seen from Figure 13a that the orientation of the layer bands is basically the same. Massive recrystallized grains can be seen in the deformation bands with high-strain energy storage and at the grain boundaries. Regions A, B and C in Figure 13b represent recrystallization, substructure and deformation region respectively. The whole region is mainly composed of LAGBs, accounting for 71.6%. The amount of LAGBS indicates that there are still a lot of substructures and dislocations in the deformed matrix. Figure 13d shows the distribution of the overall texture components. The different locations provide preferred nucleation sites for recrystallization. Texture types are represented by the colors corresponding to the corresponding color bars. The deformation band is mainly composed of Brass, Copper and S texture. The recrystallized grains in the annealed samples appear in specific deformation bands, which indicates that the nucleation position of recrystallized nuclei has obvious selectivity. The regions where the Cube{001}<100> texture, Cube-RD{120}<001> texture and BR{236}<385> texture are located are highlighted. Cube texture mainly nucleates in Copper/S deformed matrix, BR texture mainly forms recrystallized grains in Brass deformed matrix and Cube-RD texture mainly nucleates in Brass matrix. In addition, there are recrystallized grains with similar deformation orientation, which can be attributed to the orientation nucleation theory proposed by Burgers [28].

**Figure 14:**
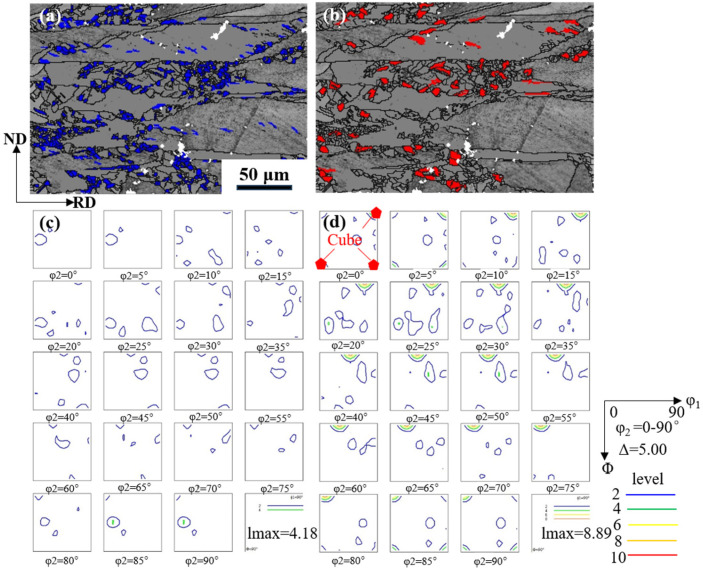
ODF map of recrystallized grains of different sizes in annealed samples: (**a**) small grain; (**b**) large grain; (**c**) ODF map of the grains marked in blue from (**a**); (**d**) ODF map of the grains marked in red from (**b**).

Figure 14 shows the orientation of large and small recrystallized grains in annealed samples. The recrystallized grains in the sample are divided into large and small grades; 7.1 μm (average grain size) ≤ large grain size ≤ 10 μm (recrystallized grains), greater than 1 μm and less than 7.1 μm are considered small grains, and large recrystallized grains are considered to be nucleated. The small recrystallized grains are post-nucleated. The large and small recrystallized new grains are highlighted in blue and red, respectively, and the other grains are shown in gray (white indicates unparsed areas). Figure 14c–d shows the ODF (Orientation Distribution Function. ODF) map of large and small grains, and it can be seen that the ODF map l_max_ of large grains is larger than that of small grains. The orientation of the large recrystallized grains in the sample is concentrated in the Cube texture (Figure 14d). In addition, a large reduction leads to further fragmentation of the grains and grain boundaries, resulting in many new nucleations of grain boundaries during recrystallization, which is conducive to the development of cube texture.

**Figure 15:**
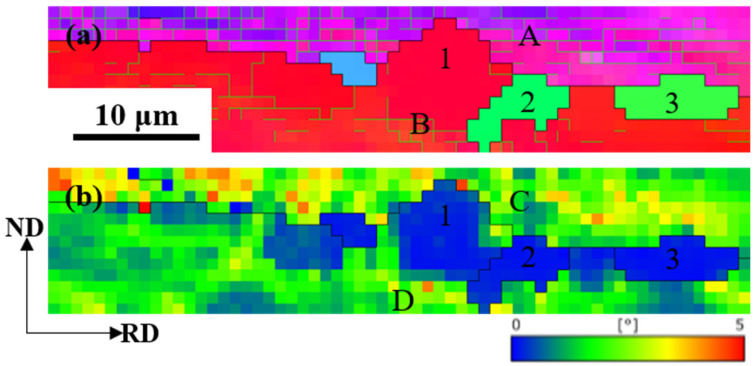
Orientation map of Cu-Ni-P alloy annealed at 500 °C for 30 min: (**a**) IPF//RD map; (**b**) KAM map (1 represent deformed grain; 2, 3 represent recrystallized grain, respectively; region A, B, C and D represent the two sides of the grain, respectively).

Figure 15 shows the orientation map of Cu-Ni-P alloy annealed at 500 °C for 30 min. Recrystallized grains are formed at grain boundaries by SIBM (Strain Induced Boundary Migration, SIBM). This theory was first reported by Beck and Sperry (1950) and has been observed in various metals [29,30]. SIBM is a process in which a portion of the original grain boundary is arched out, leaving an area with a low dislocation content after migrating the grain boundary, as shown in Figure 15b. Many LAGBs are often observed in partially recrystallized microstructures. The misorientation within the “honeycomb” structures is small, but the misorientation between the other “honeycomb” structure is large, as shown by the black line in Figure 15a, forming HAGBs. The KAM (Kernel Average Misorientation, KAM) map is calculated using the nearest neighbor points (a 3 × 3 region) and the threshold is set to 5°. The KAM value of region C is relatively higher than that of region D. In general, grain boundaries arc from the low-energy region to the high-energy region. Grain 1 gradually erodes the surrounding deformed grains to form recrystallized grains such as grains 2 and 3 as the annealing continues.

**Figure 16:**
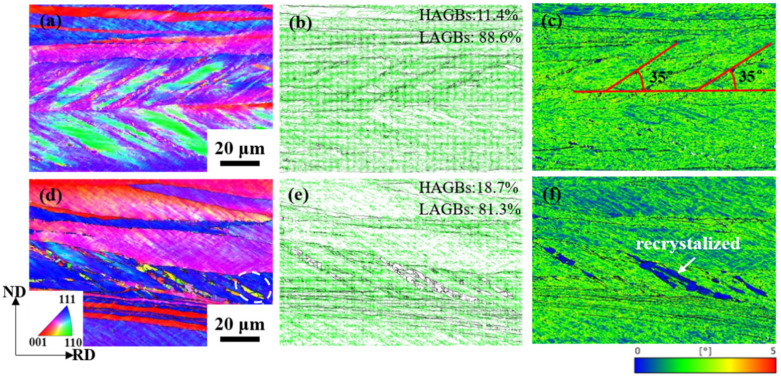
Orientation map of Cu-Ni-P alloy with 80% rolling reduction after cold rolling and annealing at 500 °C for 5 min: (**a**,**d**) IPF map; (**b**,**e**) grain boundary map; (**c**,**f**) KAM map.

Figure 16 shows the orientation map of Cu-Ni-P alloy with 80% cold rolling reduction and annealing at 500 °C for 5 min. The KAM map was used to reflect the local strain of the sample. The proportion of LAGBs of Cu-Ni-P alloy after cold rolling is 88.6% and massive LAGBs evolved from the dislocation wall formed by the accumulation and rearrangement of dislocations during cold rolling as shown in Figure 16b. The higher the KAM value, the greater the local plastic strain and the higher the dislocation density. Therefore, the density of LAGBs also reflects the dislocation density of the deformed structure (Figure 16c) [31]. The shear band was observed on the ND-RD surface and was measured to be ~35° in the rolling direction. Shear bands in rolled metal are thin regions of high-strain material, and they are usually the result of strain inhomogeneity during rolling [32]. As mentioned above, a small amount of LAGBs is transformed into HAGBs by rotation during the rolling process, which is mainly distributed near the primary grain boundary of the deformed structure. After annealing at 500 °C for 5 min, the dislocation density of Cu-Ni-P alloy decreases due to the recovery process during heating, so the LAGBs distributed in the deformed structure have been reduced compared with the rolled state. The release of deformable energy storage caused by annealing transforms massive LAGBs into HAGBs in the shear band, so the proportion of HAGBs increases (Figure 16e). Recrystallized grains are generated in the sample, and the recrystallization region is a low-energy region without dislocation (Figure 16f). Recrystallization occurs preferentially in the shear band except for a small amount of new grains formed at the grain boundaries, but rarely grows in the matrix between the shear bands. No recrystallization grains occurred in the matrix region at this stage.

**Figure 17:**
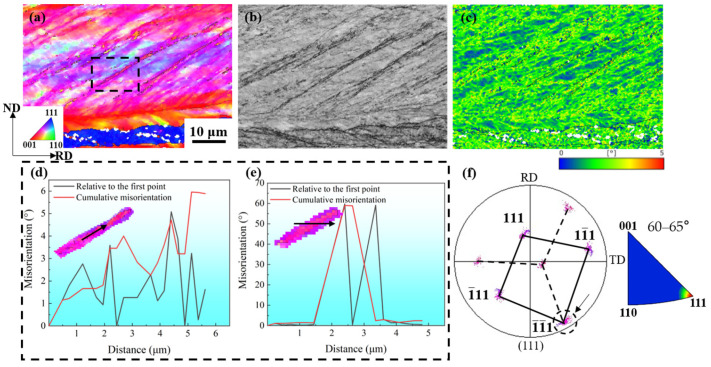
Orientation map of cold-rolled Cu-Ni-P alloy samples with 95% reduction: (**a**) IPF//RD map; (**b**) BC map; (**c**) KAM map; (**d**) misorientation angle map; (**e**) misorientation angle map; (**f**) corresponding (111) pole figure.

Figure 17b band contrast map shows that the contrast of the shear band is darker than the surrounding color. Combined with the KAM map (Figure 17c), it indicates that the dislocation density accumulated at these locations is high, which is the preferred location for recrystallization grain nucleation during annealing. Figure 17d shows the distribution of misorientation along the lines shown. At a distance of about 5 µm, the cumulative misorientation across the shear band and the local misorientation does not exceed 6°. An increase in both mutations can be detected at positions near 2.5 µm and 4.5 µm of the cumulative misorientation map. The peak value can also be observed at the abrupt position of the local orientation map. Even if the cumulative/local misorientation does not exceed 10°, however, several jumps and fluctuations in the misorientation mean that there is an orientation gradient, which would be a favorable nucleation site for recrystallization. This may cause the recrystallized grains formed in the shear band to grow only along the shear band and rarely grow in the matrix part between the shear bands [33]. Figure 17e shows the distribution of misorientation along the lines shown. It can be seen that there is an approximate 60° misorientation between the shear band and the surrounding matrix, and the misorientation axis is 111. The distance between the two peaks of the point-to-point misorientation corresponds to the width of the shear band, which is about 1.5 μm. The (111) pole figure of Figure 17f shows that the shear band is twinning with the matrix, and its twin plane is ($\overset{-}{1}\overset{-}{1}1$). The twins produced by the FCC structure metal during deformation are {111}<112>. This may be the reason why the rolling direction in Figure 6(e2) finally forms <112>.

**Figure 18:**
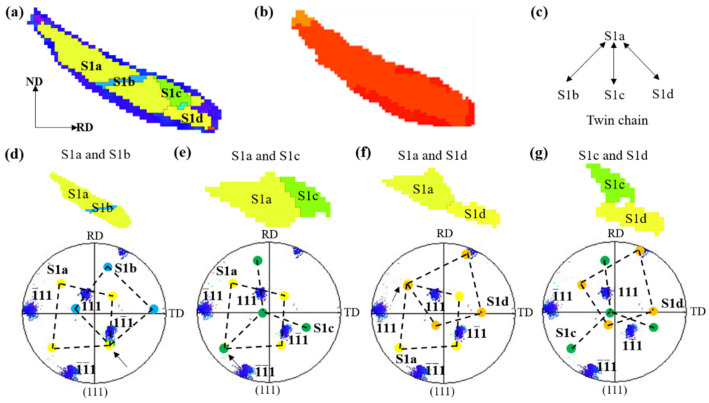
Local orientation map of Cu-Ni-P alloy with 80% reduction after annealing at 500 °C for 5 min: (**a**) IPF//RD map; (**b**) TRD map; (**c**) twin chain; (**d**–**g**) (111) pole figure.

Figure 18 shows the nucleation of multiple grains in the shear band. The orientation relationship of recrystallized grains in the shear band is further studied. In the early stage of recrystallization, recrystallization occurred only in the shear band, but the recrystallized grains did not grow into the matrix region between the shear bands [34]. Figure 18b reconstructed the orientation map of Figure 18a, and the selected grains generally had twin relations. An analysis of the orientation shows that the grain contains twin chains of different lengths, as shown in Figure 18c. Each bidirectional arrow in Figure 18c defines a pair of twin relationships. The (111) pole figure shows the optimal distribution of twin-oriented grains and deformed matrix (Figure 18d–f), and the colors of different solid circles in the pole figure correspond to the orientation coloring of Figure 18a. The black arrow in the pole figure represents the common {111} twin plane of the two grains with twin orientation.

The recrystallization nucleation mechanism of Cu-Ni-P alloy at a 60% cold rolling reduction is mainly the bow nucleation mechanism. Shear bands begin to form in the sample when the cold rolling reduction is increased to more than 80%. The shear bands become the preferred nucleation position of recrystallization grains and continue to grow in the shear bands during the annealing process. Most of the adjacent recrystallization grains growing in the shear band have twin relations.

## 5. Conclusions

Cu-Ni-P alloy was prepared by vacuum melting in this paper. The microstructure and texture evolution of Cu-Ni-P alloy during cold rolling and annealing at 500 °C was studied. The following conclusions can be drawn:

1. The equiaxed crystals formed by hot rolling of Cu-Ni-P alloy are elongated and the dislocation density increases with the increase in the reduction. The random orientation of grain decreases gradually, and the texture strength increases, and four typical textures of Goss, Brass, S and Copper are formed.
2. The deformation structure gradually disappeared and recrystallized new grains were formed with the increase in the annealing time with the annealing at 500 °C. It is found that Cube texture is mainly nucleated in Copper-oriented deformed grains, and BR texture is mainly formed in Brass oriented deformed grains after 60% cold rolling +500 °C/30 min.
3. The recrystallization nucleation mechanism of Cu-Ni-P alloy at 60% cold rolling is mainly the arch nucleation mechanism. Shear bands begin to form in the sample when the reduction in cold rolling is increased to more than 80%. The shear band becomes the preferred nucleation position of the recrystallized grains and continues to grow in the shear band in the annealing process. Most of the adjacent recrystallized grains growing in the shear band have twin relations.

## Figures and Tables

**Table 1:** Chemical composition of the as-received sample.

Element	Ni	P	Cu
nominal (wt.%)	0.600	0.158	Bal.
(wt.%)	0.584	0.165	Bal.
(wt.%)	0.611	0.174	Bal.
(wt.%)	0.607	0.162	Bal.
(wt.%)	0.595	0.170	Bal.
Standard deviation	0.0106	0.006	

## Data Availability

The raw data supporting the conclusions of this article will be made available by the authors on request.
